# Isolation of Patients With Bacterial Pneumonia Suspected of COVID-19 Leads to Prolonged Hospitalization

**DOI:** 10.7759/cureus.32155

**Published:** 2022-12-03

**Authors:** Akiko Tamura, Manabu Murakami, Torahiko Jinta, Hiroshi Okamoto

**Affiliations:** 1 Respiratory Medicine, National Center for Global Health and Medicine, Tokyo, JPN; 2 Department of Respiratory Medicine, St. Luke's International Hospital, Tokyo, JPN; 3 Department of Critical Care Medicine, St. Luke's International Hospital, Tokyo, JPN

**Keywords:** isolation, oxygen therapy, hospitalization, bacterial pneumonia, covid-19

## Abstract

Objective

The coronavirus disease 2019 (COVID-19) pandemic has changed the inpatient treatment practice for bacterial pneumonia. Upon hospitalization, isolation is required while waiting for the polymerase chain reaction (PCR) test result, which may lead to limited access to medical resources and fewer room visits by medical staff. However, little is known about the relationship between isolation and the clinical outcomes of bacterial pneumonia. We hypothesized that isolation of suspected COVID-19 patients who are eventually diagnosed with bacterial pneumonia is associated with a prolonged length of hospitalization as compared with non-isolation.

Patients

This is a single-center, retrospective observational study of hospitalized adult patients diagnosed with bacterial pneumonia from January 2018 to September 2021. The patients were divided into a non-isolated (patients hospitalized between January 2018 and December 2019, who were not isolated at all) and an isolated group (patients hospitalized between January 2020 and September 2021, who were initially isolated because COVID-19 was suspected). The primary outcome was a prolonged length of hospitalization (≥14 days), and its relationship with isolation was analyzed using logistic regression analysis adjusted for age, sex, CURB-65, and the Charlson Comorbidity Index.

Results

Among 917 eligible patients, 214 (23%) underwent isolation. In the logistic regression analysis, the isolated group independently had a prolonged length of hospitalization as compared with the non-isolated group (odds ratio 1.49; 95% confidence interval 1.08-2.07, p=0.015). There was no significant difference in antibiotic use duration between the groups.

Conclusion

The isolation of bacterial pneumonia patients suspected of COVID-19 was associated with prolonged length of hospitalization.

## Introduction

Since its outbreak in December 2019, the coronavirus disease 2019 (COVID-19) pandemic has changed inpatient treatment practices for bacterial pneumonia. Currently, patients with bacterial pneumonia are isolated on admission until at least one negative polymerase chain reaction (PCR) test because their symptoms, such as fever and cough, are indistinguishable from those of COVID-19 [1]. Patients are placed in a single-occupancy room, and COVID-19 guidelines recommend minimizing the frequency of entry into the room, which may limit access to medical resources such as regular nurse care and physiotherapy interventions [2-4]. However, little is known about the relationship between isolation and the clinical outcomes of bacterial pneumonia. We suspected that isolation negatively affects patients and hospitals because it raises the bar for examinations and treatment as well as reduces room visits by medical staff, especially nurses, certified nursing assistants, and physical therapists/occupational therapists. One marker for negative impact is prolonged length of hospitalization because it can cause physical and mental impairment in patients as well as reduce the number of available beds for others. Therefore, we hypothesized that isolation is associated with prolonged hospitalization in patients primarily suspected of COVID-19 who are eventually diagnosed with bacterial pneumonia compared to that in those with pre-pandemic, non-isolated bacterial pneumonia.

This article was previously presented in a poster session at the European Respiratory Society International Congress 2022 on September 6, 2022.

## Materials and methods

This single-center, retrospective observational study was conducted at St. Luke’s International Hospital, a tertiary care center and designated medical facility for COVID-19 admission. The study protocol was approved by the ethics committee of St Luke's International Hospital (approval number 21-J026). The need for written informed consent was waived because of the retrospective design and the minimal risk to participants. Hospitalized adult patients (aged ≥18 years) diagnosed with bacterial pneumonia from January 2018 to September 2021 were included.

Patients diagnosed with bacterial pneumonia in this context were defined as those who received antibiotic therapy from the first day of hospitalization, with an administrative coding of “bacterial pneumonia,” “aspiration pneumonia,” or “acute pneumonia.”

We excluded cases of COVID-19 overlap, early transfer to another hospital (≤five days after admission), discharge against medical advice, isolation for reasons other than COVID-19 suspicion (e.g., tuberculosis), and cases where patients were isolated for < one day.

Patients were then dichotomized into non-isolated; hospitalized before the pandemic, and isolated; hospitalized after the pandemic groups.

Isolation was defined as a situation whereby patients are kept in a single-occupancy room on admission, with the medical staff required to use personal protective equipment. In deciding the duration of isolation, clinicians evaluated the likelihood of COVID-19 overlap by considering CT findings and recent exposure to COVID-19. When the likelihood of COVID-19 appeared low, only one negative result of the polymerase chain reaction (PCR) test for SARS-CoV-2 was required to terminate the isolation; however, when the likelihood appeared high, multiple PCR tests on different days were required.

The primary outcome was a prolonged length of hospitalization (≥ 14 days), and secondary outcomes were longer duration of oxygen therapy (≥ 14 days), antibiotic therapy (≥ seven days), risk of death, and risk of intensive care unit (ICU) admission. These definitions were based on the Japanese medical fee system according to which the first 14 days of hospitalization are regarded as the acute phase, during which hospitals receive government subsidies and are encouraged to discharge quickly [5]. The definition of a longer duration of antibiotic therapy was based on recent guideline recommendations [6]. Patients were given oxygen therapy based on the oxygen saturation (SpO2) level of pulse oximetry. In most cases, oxygen therapy started when the SpO2 was below 92%. In cases where the patients had underlying lung comorbidities, such as COPD, the minimum requirement of SpO2 was decreased to 88-92%.

For statistical analysis, we compared patient characteristics and outcomes between the isolated and non-isolated groups using the χ2 or Mann-Whitney U tests, as appropriate. To determine the association between isolation and patient outcomes, we fitted logistic regression models adjusting for age, sex, CURB-65, and the Charlson Comorbidity Index. These covariates were selected based on a priori knowledge and clinical plausibility [7,8]. Statistical significance was defined as P <0.05. All analyses were performed using Easy R (Saitama Medical Center, Jichi Medical University, Japan), a graphical user interface for R (The R Foundation for Statistical Computing, Vienna, Austria) [9].

## Results

**Figure 1:**
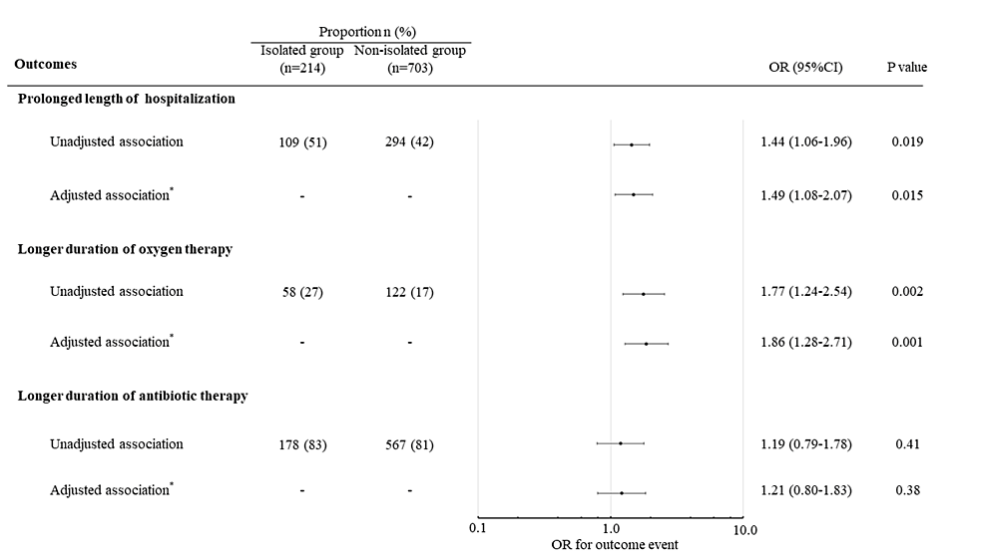
Unadjusted and adjusted associations between isolation and prolonged length of hospitalization (≥ 14 days), longer duration of oxygen therapy (≥ 14 days), and longer duration of antibiotic therapy (≥ seven days) CI, confidence interval; OR, odds ratio *Logistic regression model adjusted for age, sex, Charlson Comorbidity Index, and CURB-65 score

In total, 1,093 patients were diagnosed with bacterial pneumonia. We excluded four patients with COVID-19 overlap, 19 patients with an early transfer to another hospital, one patient with self-decided discharge, two patients with isolation for reasons other than COVID-19 suspicion, and 150 patients with isolation < one day. Of the 917 eligible patients, 214 (23%) were isolated and 703 (77%) were not (Table 1). The median length of isolation was three days. The median of CURB-65, the severity index for pneumonia, was not different between the two groups (3 points vs. 3 points; P=0.36). Isolation was independently associated with prolonged length of hospitalization (odds ratio (OR), 1.49; 95% confidence interval (95% CI), 1.08-2.07; P=0.015) (Figure 1), and longer duration of oxygen therapy (OR, 1.86; 95% CI, 1.28-2.71; P=0.001), but not with longer duration of antibiotic therapy (OR, 1.21; 95% CI, 0.80-1.83; P=0.38). There was no significant difference between the two groups for risk of death (OR, 0.99; 95% CI, 0.59-1.66; P=0.96) or ICU admission (OR, 0.66; 95% CI, 0.36-1.21; P=0.18).

**Table 1:** Patient characteristics and outcomes Patient characteristics are described using median and interquartile range (IQR) for continuous variables and number and percentage (%) for categorical variables. IQR, interquartile range

Variables	Non-isolated group (n=703)	Isolated group (n=214)	P-value
Age (year), median (IQR)	84 (76, 90)	83 (76, 89)	0.43
Male sex	385 (54.8%)	133 (62.1%)	0.059
Charlson comorbidity index, median (IQR)	7 (5, 10)	6 (4, 8)	<0.001
*Curb-65*, median (IQR)	3 (2, 4)	3 (2, 4)	0.36
Isolation period (day), median (IQR)	0.0 (0.0, 0.0)	3.0 (2.0, 5.0)	<0.001
Length of hospitalization (day), median (IQR)	11.0 (8.0, 19.5)	14.0 (8.0, 24.8)	0.017
Duration of oxygen therapy (day), median (IQR)	3.0 (0.0, 9.0)	5.5 (1.25, 15.8)	0.001
Duration of antibiotic therapy (day), median (IQR)	10.0 (7.0, 15.0)	10.0 (7.25, 18.0)	0.12

## Discussion

Although there are many studies on the prognosis of COVID-19, few have investigated the prognosis of non-COVID-19-related diseases [10,11]. This study is unique in that it focused on the impact of the pandemic on patients with bacterial pneumonia, a leading cause of hospital admissions worldwide [12]. Our results indicated that isolation was independently associated with a prolonged length of hospitalization, and we suspect that this may be due to the longer duration of oxygen therapy. In infectious diseases that necessitate isolation, such as tuberculosis and human immunodeficiency virus, the quality of nursing care is crucial since nurses are the front-line care providers [13]. This was also true for COVID-19, and after the pandemic, the barriers to providing good care for patients have been discussed [14]. Isolation reduces the opportunity for nursing care, such as position changing, suction of sputum, and adjustment of oxygen devices, which are essential for reducing oxygen demand [15,16]. Combined with our result, we speculated that a longer duration of oxygen demand ultimately led to a prolonged length of hospitalization. Because there was a gap between the median duration of hospital stay and oxygen therapy (14.0 days vs 5.5 days), we believe that the longer duration of oxygen therapy indirectly affected the length of hospital stay, as oxygen therapy during the first few days and respiratory distress negatively affected physical activity and eating, leading to a decline in activities of daily living and a longer time to recovery. However, isolation was not associated with a longer duration of antibiotic therapy because the standard duration for antibiotic therapy is five to seven days as per guidelines [6]. In addition, complete oxygen weaning is not necessarily a prerequisite for ending antibiotic treatment; increased sputum production or atelectasis are commonly observed in patients with late-phase bacterial pneumonia, even after the inflammation has peaked out.

As a result, in a situation where access to nursing care is limited, there is a need to shorten the length of oxygen therapy to avoid the prolonged length of hospitalization. One method of shortening the length of oxygen therapy is to simply ask patients to lie on one side and then on the other side after several hours, to promote sputum drainage [15]. Additionally, in the case of patients with cognitive impairment and dysphagia, continuous oral suctioning of saliva can be beneficial for preventing aspiration and deterioration of pneumonia [16]. Further studies are required to investigate whether these methods are effective in reducing the duration of oxygen therapy and hospitalization.

Although the length of the isolation period is becoming shorter than that at the beginning of the pandemic due to increasingly efficient PCR testing, this research can still be applied in a situation where the clinicians estimate that the likelihood of COVID-19 is high from the CT findings and where the patients have contact with COVID-19 patients because in these cases, PCR tests are often repeated twice or three times on a different day and isolation lasts for more than two or three days. These findings will also be helpful if a new pandemic that necessitates isolation occurs.

This study’s limitations were as follows: it was a single, retrospective study, and other factors that can extend the length of hospital stays, such as episodes of delirium, were not evaluated. The information about whether a patient was hospitalized during the COVID-19 pandemic lockdown was not available although this information can affect factors such as length of isolation. Although all the included patients received antibiotic therapy, they might have been infected with non-COVID-19-related viral pneumonia since we did not exclude patients whose bacterial culture test was negative. Also, since the background etiology of aspiration pneumonia is different from other types of pneumonia, it might be more appropriate not to include it. However, we decided to include it in this study because the median age of our study participants was as much as 83-84, and from an individual chart review, many cases of aspiration pneumonia were indistinguishable from those of acute pneumonia. In addition, we performed multiple logistic regression analyses because our study's aim was to assess whether the length of hospitalization exceeded the acute phase defined by the government health insurance system, which was 14 days. However, outside Japan, the length of hospitalization tends to be shorter, and healthcare systems are often different; therefore, this result may not apply to other countries.

## Conclusions

Isolating patients with bacterial pneumonia suspected of COVID-19 is associated with a prolonged length of hospitalization, and this may be due to longer oxygen therapy. However, isolation is not associated with a longer duration of antibiotic therapy. Isolated patients may be deprived of the opportunity for proper nursing care, including position changing and sputum suctioning, which are important for reducing oxygen demands; this may ultimately result in a prolonged length of hospitalization. Therefore, new methods for reducing oxygen demand without the need for nursing care should be sought.

In a new pandemic, such as COVID-19, isolation of patients with diseases of similar symptoms is sometimes inevitable. However, physicians should be mindful of the negative impact of isolation and should consider ways of avoiding unnecessary isolation, as well as making efforts to maximize patient care without increasing the burden of other healthcare professionals.
